# Advancements and Innovative Strategies in Induced Pluripotent Stem Cell-Derived Mesenchymal Stem Cell Therapy: A Comprehensive Review

**DOI:** 10.1155/2024/4073485

**Published:** 2024-09-30

**Authors:** Xiaoyu Shi, Kun Zhang, Fengshi Yu, Qi Qi, Xiaoyu Cai, Yu Zhang

**Affiliations:** ^1^ State Industrial Base for Stem Cell Engineering Products, Tianjin 300384, China; ^2^ VCANBIO Cell and Gene Engineering Corp. Ltd., Tianjin, China

## Abstract

The effectiveness and safety of mesenchymal stem cell (MSC) therapy have been substantiated across various diseases. Nevertheless, challenges such as the restricted *in vitro* expansion capacity of tissue-derived MSCs and the clinical instability due to the high heterogeneity of isolated cells require urgent resolution. The induced pluripotent stem cell-derived MSCs (iPSC-MSCs), which is differentiated from iPSCs via specific experimental pathways, holds considerable potential as a substitute for tissue derived MSCs. Multiple studies have demonstrated that iPSCs can be differentiated into iPSC-MSCs through diverse differentiation strategies. Research suggests that iPSC-MSCs, when compared to tissue derived MSCs, exhibit superior characteristics in terms of proliferation ability, immune modulation capacity, and biological efficiency. In this review, we meticulously described and summarized the experimental methods of iPSC differentiation into iPSC-MSCs, the application of iPSC-MSCs in various disease models, the latest advancements in clinically relevant iPSC-derived cell products, and the development strategies for the next generation of iPSC-derived therapy products (not only cell products but also their derivatives).

## 1. Introduction

Regenerative medicine and cell therapy have emerged as central areas in the realm of advanced biomedical technologies globally. In this domain, the potential of mesenchymal stem cell (MSC) therapy has been substantiated, showing limitless applications and demonstrating safety and efficacy in clinical trial research across diverse diseases, thereby eliciting substantial interest.

MSCs derived from various tissues, exhibit functional characteristics encompassing immune modulation, anti-inflammatory effects, hematopoietic function supporting, and tissue repair capabilities [1]. Their utility extends to treating autoimmune diseases such as systemic lupus erythematosus, inflammatory conditions such as osteoarthritis, age-related disorders, and infectious diseases [2]. Nevertheless, the distinct MSC cell sources, donor condition variations, and culture system differences resulted in cell heterogeneity, restricted *in vitro* expansion capacity, and challenges in cell isolation and extraction [3]. Thus, MSC cell therapy has some limitations and unstable effects in clinical application [4, 5].

In response to this challenge, an increasing number of researchers are investigating the utilization of pluripotent stem cells (PSCs) as original cells, prompting their differentiation into MSCs for diverse diseases. There are two kinds of PSCs, namely embryonic stem cells (ESCs) and induced pluripotent stem cells (iPSCs), possessing unlimited self-renewal and differentiation potential [6]. ESCs, primarily sourced from human embryos and evoke potential ethical concerns [7]. In 2006, Shinya Yamanaka pioneered the reprogramming of differentiated somatic cells into PSCs using Sendai virus-mediated delivery of four transcription factors (OCT4, SOX2, KLF4, and c-Myc). This groundbreaking discovery prompted scientists worldwide to embark on extensive research on iPSCs, marking the onset of the iPSC era [8, 9, 10]. iPSCs have diverse origins, obtainable from various mature cell types, including epithelial fibroblasts [9, 11], adipocytes [12], renal epithelial cells [13], neurons [14], pancreatic islet cells [15, 16], mesenchymal stem cells [17], and renal epithelial cells [18]. iPSCs retain some epigenetic markers from the donor cells, which may influence the cell function in various differentiation directions [19, 20, 21].Conversely, iPSCs have been shown to be de-differentiated from assorted differentiated adult cells such as fibroblasts and peripheral blood cells [22]. In comparison to tissue-derived MSCs, iPSC-derived cell populations from the same cell line can undergo limitless *in vitro* expansion while preserving pluripotency, ensuring a stable phenotype, and biological potency. They exhibit a uniform quality, a singular source, and convenient procurement, thereby sidestepping ethical issues linked with the utilization of human ESCs [23].

This review delineates the methodological research progress in differentiating iPSCs into iPSC-MSCs, the application of iPSC-MSCs in diverse disease models, the latest research advancements in clinically relevant iPSC-derived cell products and the developmental strategies for the subsequent generation of iPSC-derived cell therapy products and the exosome products. This intends to enhance product development strategies, facilitating the maximization of the potential and advantages of iPSCs in tissue engineering and personalized cell therapy.

## 2. Methodological Research in Obtaining iPSC-MSCs

**Figure 1:**
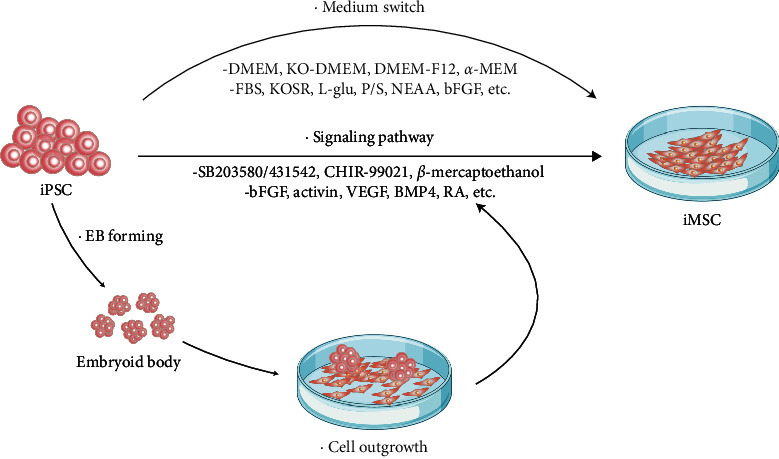
iPSC to iMSC differentiation process flowchart. The predominant routes for iPSC to iMSC differentiation include MSC culture medium substitution induction, induction of iMSC formation through signal pathway modulators, and embryoid body formation.

There are many prevalent methods reported for preparing iPSC-derived MSCs (iMSCs) [24]. Differentiation approaches can be broadly categorized into three aspects with mainstream strategies, including MSC culture medium substitution induction, signal pathway regulation, and embryoid body (EB) forming methods [25]. We have summarized these methods in Figure 1. In the subsequent sections, we provided detailed descriptions of the key materials and methodological details involved in each reported research. Each study evaluates the cell surface markers and multipotent differentiation potential of the iMSCs. Additionally, we presented statistical data of time cost for overall differentiation approaches, aiming to identify a safe and efficient differentiation scheme suitable for industrial-scale production.

### 2.1. MSC Culture Medium Substitution Induction

By substituting iPSC culture medium with an MSC culture system, spontaneous differentiation of iPSCs can be induced, serving as an induction and screening mechanism for cells. The basic components of MSC culture medium include high/low glucose dulbecco's modified eagle medium (DMEM), KO-DMEM (knockout specific component with DMEM), DMEM-F12, and *α*-MEM, supplemented with fetal bovine serum (FBS), knockout serum replacement (KOSR), L-glutamine, penicillin/streptomycin (P/S), nonessential amino acids, and fibroblast growth factor (FGF).

Kang et al. [26] and Lang et al. [27] reported that replacing iPSC culture medium with DMEM containing 10% FBS for 2 weeks, followed by continuous passaging in precoated culture dishes, yielded iMSCs with a fibroblast-like morphology. Similar induction conditions using *α*-MEM medium [28] also proved effective. However, the overall differential efficiency of this method is relatively low. To obtain iMSCs with a qualified phenotype (high expression of CD73, CD44, CD105, and CD90; low expression of CD19, CD34, CD45, and HLA-DR); trilineage differentiation capacity [29, 30], the process takes an extended period of 30–50 days.

### 2.2. Induction of iMSC Formation by Signal Pathway Modulators

Pathway inhibition is a commonly employed method for inducing iPSCs into iMSCs, swiftly and efficiently attaining MSC cell phenotype with functionality. Inhibitors used for iPSC-MSC induction include SB203580 (p38-MAPK inhibitor), SB431542 (TGF-*β* inhibitor), CHIR-99021 (GSK3 inhibitor), *β*-mercaptoethanol, and bFGF.

### 2.3. SB203580

SB203580 is a pyridinyl imidazole-type p38 MAPK inhibitor, demonstrating inhibitory activity against kinases such as GAK, CK1, RIP2, c-Raf, and GSK3. The p38-MAPK pathway plays a role in promoting MSC differentiation into epidermal-like cells [31]. Additionally, it can inhibit IL-2-mediated T-cell proliferation and participate in various inflammatory responses or induce cellular autophagy *in vivo*. Studies have reported that SB203580 can facilitate the transformation of iPSCs towards a mesodermal phenotype [32]. It involves the formation of EBs from suspended iPSCs, inducing EBs with a specific concentration of SB203580 in the culture medium. The induced EBs are then passaged in adherent culture, resulting in iPSCs with qualified surface markers after 28 days. Moreover, even after 20 passages, the cells exhibit intact karyotypes and show no properties of aging.

### 2.4. SB431542

SB431542 is a TGF-*β* signaling pathway inhibitor, and the TGF signaling pathway is a key pathway for maintaining stem cell pluripotency. Inhibiting the TGF pathway can induce ESCs and iPSCs to differentiate into MSCs or neuronal cells [33]. Glaeser et al. [34] utilized 20 *μ*M SB431542 for 10–14 days to sequentially induce ESCs into neural crest cells, followed by differentiation into MSC-like cells in a serum-containing culture system. Kim et al. [35, 36], Sun et al. [37], and Chen et al. [38] added 10 *μ*M SB431542 throughout the entire iMSC induction process, obtaining iMSC lines with trilineage differentiation capability after several passages. The concentration of SB431542 used by different researchers ranged from 1 to 20 *μ*M [39], with no significant differences observed in the induction efficiency of iMSCs.

### 2.5. CHIR-99021

CHIR-99021 is a highly specific glycogen synthase kinase-3 (GSK-3) inhibitor, proven to promote self-renewal and maintain pluripotency of ES cells in BALB/c mouse [40]. CHIR-99021 is involved in various signaling pathways, including Wnt/*β*-catenin, TGF-*β*, nodal, and MAPK [41]. Different concentrations of CHIR99021 are often used in combination with SB431542 for inducing iMSC differentiation. Harada et al. [42] and Fukuta et al. [43] employed a compound combination of 10 *μ*M SB431542 with 1 *μ*M CHIR99021, successfully inducing mesenchymal phenotype cells following the iPSC-neural crest cell-MSC-like cell route in about 21 days. Winston et al. [44] used 4 *μ*M SB431542 and 4 *μ*M CHIR99021 with 10 ng/mL bfgf to obtain iMSCs in approximately 25 days. The addition of signaling pathway modulators, when compared to direct conversion with MSC culture medium, enhances the efficiency of inducing mature iMSCs, significantly shortening the overall differentiation time. Moreover, the obtained cells show no apparent differences from primary MSCs in terms of phenotype, trilineage differentiation capacity, and genome expression [7].

### 2.6. *β*-Mercaptoethanol

*β*-mercaptoethanol is a frequently used additive in the induction process of iMSCs. Many researchers incorporated varying concentrations of *β*-mercaptoethanol into iPSC culture systems (final concentrations of 55 *μ*M [45], 0.1 mM [46, 47], 110 mM [48]). After approximately 30 days, fibroblast-like iMSC cells were obtained, and upon identification, the cell surface markers and trilineage differentiation capacity were found to be similar to bone marrow-derived MSCs (BM-MSCs).

### 2.7. Embryoid Body Method

The differentiation pathway facilitated by EBs in iMSC induction allows for the harvesting of more target cells within a limited culture volume, facilitating industrialization and cost control. However, the overall cultivation process requires a longer time, and the harvested cells exhibit a certain degree of heterogeneity. Researchers typically introduce compound pathway modulators into the culture system during the formation of EBs and the emergence of iMSCs to enhance the efficiency of cell enrichment towards iMSCs.

Chen et al. [38] employed DMEM-F12 medium containing 20% KOSR and 0.1 *μ*g/mL bFGF to cultivate iPSCs in suspension, forming EBs. Subsequently, 10 *μ*M SB431542 was introduced into the system to promote cell emergence, followed by cell passaging to obtain iMSCs. Li et al. [49] developed a method for the largescale expansion of ESCs in a bioreactor, generating a substantial number of EBs through ESC suspension culture. They induced EB differentiation using a system containing KOSR, followed by further induction to promote EB emergence, resulting in ES-MSCs. Researchers further optimized the induction system, creating a novel formula by adding specific concentrations of bFGF and TGF*β*-1 to the system to enhance the emergence and maturation of ES-MSCs [50]. Additionally, various cytokines, including activin A [51], vascular endothelial growth factor (VEGF), BMP4 [52], and RA [53], have been applied in the induction process through EB differentiation, with differentiation periods ranging from 20 to 30 days.

The selection of materials and components for the coating employed in the iMSC induction process is contingent upon varied experimental methodologies and specific requirements. Among the commonly utilized coating materials are matrigel, vitronectin, fibronectin, iMatrix 511, laminin 521, etc.

## 3. Applications of iMSCs

### 3.1. The Comparation of Function and Properties Between Primary MSCs and Derived iMSCs

Researchers have compared iMSCs induced from iPSCs derived from different sources, such as dental pulp, periodontal ligament, and lung tissue. They found that all iMSCs exhibited similar characteristics in self-renewal, multilineage potential, and surface marker expression [54]. Previous research reported that compared with the donor matched UC-MSC, iMSCs exhibited higher neural differentiation potential and showed higher immunosuppressive function compared to UC-MSC *in vitro* [55]. The Table 1 below outlines the performance characteristics of iMSCs obtained from iPSC differentiation originating from different tissues. Notably, iMSCs exhibit superior functional properties compared to adult MSCs with greater proliferative capacity, higher immunomodulatory potential, more superior bioactive paracrine secretion, and more generation of microenvironment-modulating exosomes.

### 3.2. Preclinical Research Data for Various Indication Models

iMSCs have been utilised in numerous animal disease models to showcase their therapeutic efficacy. iMSCs demonstrate functions similar to MSCs in tissue regeneration, injury repair, immune modulation, and inflammation regulation.

The crucial consideration lies in the low immunogenicity of iMSCs, whether administered via intravenous infusion or local injection. Research findings indicate that the introduction of iMSCs into the knee joint cavity of nude mice and SD rats afflicted with cruciate ligament injuries does not elicit an inflammatory response, thereby underscored is the applicability of iMSC xenotransplantation [61]. In terms of immunomodulatory functionality, the infusion of iMSCs significantly diminishes T-cell intensity, reduces post-transplant monocyte infiltration, diminishes the expression of pro-inflammatory cytokines IFN*γ* and TNF*α* in the plasma, fosters IL-10 expression, and markedly extends hind limb survival time in a rat graft-versus-host disease (GVHD) model [62]. In the context of inflammatory bowel disease, whether administered via the tail vein or intraperitoneal injection, iMSCs markedly ameliorate mouse symptoms, stimulate the proliferation of intestinal epithelial cells, and enhance intestinal vascularization, with analogous effects observed to an equivalent dose of adipose tissue-derived mesenchymal stem cells (AT-MSCs) [63].

Within the sphere of tissue regeneration, iMSC cell sheets significantly augment cartilage tissue regeneration in nude mice afflicted with laryngeal cartilage injuries, amplifying the expression of cartilage cell markers, and the deposition of extracellular matrix [7]. Concerning bone regeneration, local transplantation of iMSCs yields a markedly superior bone matrix compared to calcium phosphate particles, with no significant difference observed in the performance of BM-MSC transplantation in cortical and central defect areas [64]. In a steroid-induced rat femoral head necrosis model, the local injection of iMSCs prevents bone loss in the necrotic area and promotes cartilage repair [65]. The implantation of iMSCs into a rat periodontal defect model stimulates periodontal regeneration and the formation of new mineralized tissue [66]. Transplanting osteoblasts induced from iMSCs into mice with cranial bone defects supports bone formation at the defect site [67].

iMSCs exhibit notable efficacy in the realm of injury repair. In a rat model of cerebral ischemia-reperfusion injury, iMSCs demonstrate the capability to inhibit the activation of glial cells at the injury site, leading to a significant improvement in both short-term and long-term neurological and motor functions in rats [68]. In a mouse model of renal ischemia-reperfusion injury, varying doses of iMSCs activate the ERK1/2 signaling pathway, mitigating serum creatinine levels, and the extent of renal tubular necrosis. Additionally, they significantly decrease inflammatory cytokines and oxidative stress levels, culminating in an enhancement of renal function [48]. The local injection of iMSCs in proximity to the infarcted area in a myocardial infarction mouse model fosters interaction between parenchymal cells and stromal cells, thereby mitigating ventricular remodeling [69]. In a mouse model of passive smoking-induced cardiac dysfunction, intravenous injection of iMSCs significantly attenuates the elevation of pro-inflammatory cytokines, reinstates the expression of anti-inflammatory factors and antioxidant markers, and markedly improves abnormal cardiac morphology [70]. Furthermore, in anthracycline-induced cardiomyopathy mouse model, iMSC infusion resulted in more mitochondrial retention and bioenergetic preservation that could effectively attenuate cardiomyocyte damage [71].These findings suggest the potential of iMSCs as therapeutic agents for cardiovascular conditions.

Moreover, the application of iMSCs is extensive in addressing functional disorders. Intrasplenic injection of iMSCs in rats with liver dysfunction results in the differentiation of iMSCs into functional liver cells expressing various human-specific stem cell markers, thereby promoting liver regeneration [72]. iMSCs infusion proves effective in improving serum biochemical indicators in rabbits with renal dysfunction, reducing the degree of renal tissue fibrosis, and restoring renal blood flow [73]. In a rat model of erectile dysfunction, local injection of iMSCs into the corpora cavernosa repairs damaged endothelial and smooth muscle tissues, diminishes BAX and caspase-3 levels, increases Bcl-2 expression, thereby reversing cell apoptosis and restoring erectile function [74].

### 3.3. Advancements in the Research of Clinical-Grade iPSC-Derived Cellular Products

As of November 2023, an exploration of the clinical trials website revealed a total of 155 ongoing international clinical trials involving interventions based on iPSCs. The indications span a range of diseases, encompassing multiple myeloma, lymphoma, arthritis, GVHD, heart failure, respiratory failure, Parkinson's disease, stroke, and more. Among these trials, 23 clinical trials involve cellular products derived from iPSCs, with merely three specifically addressing iMSCs. The forefront of global progress is discernible in Cynata Therapeutics' CYP-001, designed for GVHD, severe limb ischemia and osteoarthritis. Their phase I clinical trial (NCT02923375) enrolled 16 participants with grade II–IV steroid-refractory acute GVHD (SR-aGVHD). The results manifest the safety and good tolerability of iMSCs, with no occurrence of serious adverse events. On the 100^th^ day of iMSC infusion therapy, the overall response, complete response, and overall survival rates reached 86.7%, 53.3%, and 86.7%, respectively, underscoring the substantial potential of iMSC products for GVHD treatment [75]. Castem, developed by Zephyrm Biotechnologies and derived from human embryonic stem cells, is currently recruiting for a phase I clinical trial in the United States (NCT04331613). The targeted indications include adult respiratory distress syndrome and acute lung injury caused by viral infections. The iMSC product NCR100 from Nuwacell has initiated a phase I clinical trial for knee osteoarthritis (NCT06049342) in China but is yet to commence recruitment. In China, five clinical trials are in progress for cellular products derived from iPSCs, with one utilizing iPSC-derived mesenchymal stromal cells. Specific details of these products are outlined in Table 2.

Combining the extant preclinical data with the status of clinical trial applications, it becomes apparent that the enthusiasm within the capital market for products derived from iPSCs is steadily increasing. The majority of iMSC products are currently in the nascent phases of clinical development, with their safety and efficacy aligning with the numerous therapeutic pipeline products of adult MSCs abundantly present in the market.

In the development of either MSCs or iMSCs as an advanced pharmaceutical product, the core challenge lies in the advancement of drug manufacturing processes and ensuring the robust quality control of stem cell therapeutics. Pharmaceutical enterprises must adhere to the principles of quality by design (QbD), which emphasizes that quality originates from the design phase. Grounded in scientific rigor and quality risk management, this approach begins with predefined quality objectives, placing significant emphasis on the comprehension of MSC/iMSC products, and their associated processes. Additionally, it entails the development of robust methods for the process control in accordance with the International Council for Harmonization (ICH) Q8 guidelines. Enterprise research and development personnel also need to conduct risk assessments to identify critical quality attributes (CQAs) that directly impact the safety and efficacy of the product, and to identify critical process parameters (CPPs) and develop a design space to quantify the variability of parameters' effects on quality attributes. Subsequently, a control strategy is developed to maintain process parameters to ensure product quality, followed by scaleup for process validation. Difficulties in the development of MSC/iMSC products include establishing good manufacturing practice (GMP)-compliant protocols, factors affecting raw material selection, cell expansion to product formulation, establishing quality control (QC) parameters, and ensuring quality assurance to comply with GMP standards [75]. CQAs of MSC products encompass identification, purity, biological potency, and safety. Also, study of comparison of iMSCs and primary tissue derived MSCs on rejuvenation, differentiation, and immunomodulatory capabilities still need more researches. Furthermore, in the development of iMSC products, additional attention is required to address product heterogeneity, which can be further analyzed through single-cell transcriptome sequencing method to assess different subpopulations of iMSC cells. Additionally, due to the potential tumorigenic risk of upstream iPSC seed cells, it is necessary to develop stable and highly sensitive methods for detecting residual iPSCs and unintended differentiated cell remnants to demonstrate the absence of tumorigenicity in the product [76]. Compared to MSCs derived from primary tissues, iMSC products undergo a longer period of culture and passaging *in vitro*, requiring attention to the genetic safety of iMSC products through whole-genome sequencing.

By establishing standardized quality control procedures and manufacturing standards, ensuring consistent quality and efficacy of MSCs from different sources or different differentiation pathway of iMSC cells. Thus, product consistency, reliability, and safety can be enhanced, facilitating their transition to GMP-grade clinical application products.

### 3.4. Development Strategies for the Next Generation of iPSC-Derived Therapy Products

#### 3.4.1. Genetically Modified Cell Products

The principal commercial applications of iPSCs and their derivatives encompass cell therapy, disease modeling, drug development and discovery, personalized medicine, and toxicology screening. Upon scrutiny of the research and market performance of such products, it is discernible that iMSC products not only confer advantages such as a straightforward preparation process, a consistent cell source, uniformity, and suitability for largescale production but also afford the convenience of genetic modification from the seed cells. This facilitates the creation of second-generation products derived from iPSCs. Studies have indicated that chimeric antigen receptor (CAR)-iPSCs, generated through specific gene editing of iPSCs, can act as seed cells for the stable production of CAR-NK cells [76]. In diverse therapeutic indications, distinct genes assume significantly varied roles. Specific modifications to genes in MSCs, ensuring the sustained expression of multiple genes, can markedly enhance the efficacy of MSCs. The overexpression of specific chemokine receptors on MSCs, such as CCR1 and CXCR2, significantly augments *in vivo* migration and homing capabilities, thereby amplifying their therapeutic effects. Genes such as IL-4, IL-10, TGF-*β*1, GATA-4, and CXCR4 have been reported to contribute to increased stem cell viability, thereby enhancing MSC efficacy [77]. The overexpression of VEGF in BM-MSCs promotes angiogenesis and improves outcomes in cases of cerebral infarction, elevating cell viability, and enhancing paracrine effects [78]. To emulate the effects of hypoxic preconditioning, transfecting hypoxia-inducible factor 1-alpha (HIF-1*α*) into BM-MSCs can reproduce their enhanced therapeutic effects under low oxygen exposure [79]. However, the application of genetic modifications to primary MSCs necessitates prolonged culture and screening, presenting challenges in avoiding replicative senescence, ultimately diminishing the therapeutic effects of MSCs. This approach may not be suitable for practical clinical applications and the development of cell therapy products. Therefore, the selection of suitable genes for specific indications, the performance of functional gene editing on iPSCs serving as differentiation starters, and subsequent induction of differentiation into iMSCs can assist researchers in obtaining iMSC cells with stable and low heterogeneity due to specific gene modifications. While this approach aids in developing enhanced iPSC-derived second-generation products, achieving sustained activation or knockdown of specific genes still necessitates extensive and complex preliminary validation to ensure the safety of the product.

#### 3.4.2. Potential of iMSC-Derived Extracellular Vesicles (EVs) as Advanced Therapeutics

EVs released by MSC-Exos hold significant promise in immunomodulation and tissue regeneration. The main functions of EVs include tissue repair and antifibrotic effects, offering therapeutic potential for various conditions such as knee osteoarthritis, spinal cord injury, skin wounds, fibrosis of liver, kidney, lung tissues, as well as neurodegenerative diseases [80]. A series of preclinical studies have also demonstrated that EVs derived from iPSC-MSCs (iMSC-EVs) exhibit similar biological functions to MSC-EVs and have shown promising therapeutic effects across various conditions [81].

Currently, the core challenges in the development of EVs as pharmaceuticals mainly revolve in two aspects: the heterogeneity of source cells leading to batch-to-batch variation, the feasibility of largescale production and purification. However, iMSCs derived from the same parental iPSC can breakthrough these challenges effectively. By selecting appropriate iPSC cell line in the early stages of the process and employing stable differentiation protocols, highly homogeneous iMSC populations can be obtained. Utilizing these homogeneous iMSC as the source for EVs production directly overcomes the heterogeneity inherent in primary cells, ensuring minimal batch-to-batch variation in the final product. Furthermore, employing appropriate preparation protocols for iMSC products ensures sustained and robust *in vitro* expansion capabilities of the cells. This allows for the direct amplification of upstream seed cell populations to expand scale in EVs production effectively.

In addition to the strategies mentioned above, improving the efficacy of iMSC-EVs can also be achieved by enhancing their concentration, targeting capabilities, and payload of functional molecules. Several approaches can be employed to enhance the therapeutic potential of iMSC-EVs. Cultivating iMSCs in 3D bioreactors can accelerate cell growth rates, thereby increasing the secretion of EVs. Pretreatment of iMSCs with small molecule drugs can regulate and enhance the secretion of EVs. Employing genetic modifications in iMSCs to express specific target molecules fused with proteins on the surface of EVs can enhance the loading of functional molecules [82].

Furthermore, adherence to international standards, such as ISO 24603, which outlines research specifications for EVs, is essential for regulating upstream steps in EV production. Recently, in China, two group standards have been issued: “Human pluripotent stem cell derived small extracellular vesicles” (T/CRHA 002-2021) and “Human mesenchymal stem cell derived small extracellular vesicles” (T/CRHA 001-2021). In product development, we should pay attention to key quality attributes of EVs, including: (1) key quality attributes of human MSCs used for EV production should meet specified requirements. (2) key quality parameters of EVs, including morphology, particle size, surface biomarkers, purity, immunomodulatory ability, and microbiological safety, should comply with standards. (3) detailed process controls should be implemented, including the EV isolation method documents.

As fundamental scientific research on EVs progresses and regulations governing EV production standards and quality standards continue to improve, iMSC-derived EVs are poised to demonstrate potential advantages, gradually transitioning towards GMP-grade products and applications.

## 4. Discussion

Presently, there are a total of 12 globally approved MSC products, and the therapeutic potential of MSC cell products has been substantiated through numerous clinical trials, signifying significant promise for treating various diseases. However, the inherent heterogeneity in MSCs sourced from adult origins directly contributes to the instability of clinical trial outcomes. In contrast, iMSC products derived from iPSCs, with the selection of cells in an optimal state and originating from the same iPSC clone as the seed, effectively mitigate the challenge of high heterogeneity in the final product. Simultaneously, the procurement of iPSC seeds, such as through the screening of human leukocyte antigen (HLA) monotypic homozygous population donors and the establishment of HLA-typed cell lines from such populations, holds the potential to diminish the risk of immune rejection during cell therapy.

In this paper, we highlighted diverse approaches for iMSC induction. The conventional two-dimensional (2D) methods were popular for their simplicity and cost-efficiency. However, a significant limitation lied in their inability to achieve high differentiation efficiency and functional homogeneity, hindering the simulation of the intricate three-dimensional (3D) environment *in vivo*. Moreover, continuous *in vitro* expansion can lead to cell senescence and compromised proliferative capacity, limiting cell availability. To address these challenges, 3D induction technology emerges as a promising solution. By mirroring the body's 3D milieu, 3D induction techniques significantly enhance cell differentiation efficiency and functional coherence. Furthermore, these methods facilitated largescale iMSC expansion through bioreactor usage, catering to commercialization, and clinical demands. Nevertheless, the utilization of microcarriers in 3D environments and the need for removing related auxiliary ingredients pose challenges in product commercialization.

As shown in Table 1, iPSC-MSCs exhibit superior biological characteristics, which in turn demonstrate improved therapeutic outcomes across various aspects. However, it is essential to recognize that the determinants influencing the pharmaceutical properties of iMSCs are similar to those of MSCs. In addition to manufacturing processes, clinical efficacy is a crucial factor. There is significant variability in clinical outcomes following MSC infusion, primarily due to the complex *in vivo* therapeutic mechanisms of MSCs, which remain poorly understood. While extensive *in vitro* research acknowledges MSCs' multilineage differentiation capacity and their immunomodulatory and anti-inflammatory effects through paracrine action, including the secretion of cytokines, exosomes, and microvesicles, as well as influencing cellular organelles to exert antifibrotic, injury repair, and tissue regeneration functions [83], their behavior in the human body is not yet fully clear. In clinical settings, researchers still need to establish correlations between therapeutic efficacy and biological activity indicators. Identifying key effector cells and molecules, examining pathways related to mechanisms of action, and developing analytical methods that correlate with product characterization, and clinical outcomes will significantly enhance the probability of MSCs becoming pharmaceutical products.

Concerning the genetic engineering of iPSC cells, the selection of specific key genes for modification and the induction of differentiation into MSCs, based on in-depth research into the disease mechanisms targeted for development, enables the production of iMSC cells with high uniformity, low mutation rates, and stable, specific expression of key genes.

As mentioned above, iMSC-derived EVs have demonstrated pharmaceutical potential in preclinical studies. However, challenges exist in the development process of iMSC-EVs. For instance, different methods of differentiating iPSCs into iMSCs directly alter the content of EVs, affecting their biological efficacy. Additionally, production costs and the feasibility of scale-up need to be considered early in the development process.

Reagent kits facilitating the stable induction of iMSCs are already commercially available, exemplified by STEMCELL Technologies STEMdiff™ [55] and Nuwacell® hPSC-MSC differentiation kit [84]. However, the development of an industrial-scale iMSC process under GMP standards and the establishment of corresponding quality assessment systems, particularly safety assessment systems, are becoming increasingly imperative. Presently, various countries have instituted policies stipulating quality standards and clinical filing criteria for iPSC-differentiated cell therapy products. Therefore, before iPSC and its derived cell products can be authentically applied clinically, it is crucial to institute a reliable, systematic safety assessment system and release criteria throughout the entire product manufacturing process. This encompasses residual detection of PSCs in the final product, clone formation experiments, nude mouse tumorigenicity experiments, and long-term tumorigenicity experiments in animals. These measures are essential for rigorous control over production processes and product quality. When selecting detection methods, factors such as testing costs, experimental duration, data analysis workload, and the complexity of result interpretation should be comprehensively considered, with adjustments and improvements made based on practical experience.

## 5. Conclusion

Presently, mainstream methods for iMSC preparation in the market can be categorized into three classes: induction through medium replacement, induction via signal pathway regulation, and embryo-like body formation. Each method exhibits varying induction efficiency, along with corresponding advantages and limitations. A growing body of preclinical and clinical research data indicates the significant potential applications of iMSC products and their second-generation counterparts across various clinical indications. This paper has furnished a detailed discussion of the aforementioned information, providing insights and references for the subsequent development of largescale, reproducible production processes conducive to commercialization. The establishment of more comprehensive quality testing standards for iPSC-derived cell therapy products will further expedite the commercial development and application of iMSC products.

## Figures and Tables

**Table 1:** Performance comparison of iPSCs and differentiated iMSCs from various tissue sources.

Source of iPSCs	Methods for iPSC-derived iMSCs	Advantages compared to MSCs
Pulmonary fibroblasts [56, 57]	(1) DMEM + 10% FBS + 10 ng/mL bFGF + 10 ng/mL PDGF + 10 ng/mL EGF (2) FACS sorting: CD24^−^/CD105^+^ and single cell cloning and plating	Stronger proliferative capacity; enhanced myogenic differentiation ability; better paracrine effect for stronger antioxidative stress; antiapoptotic actions

Amniotic fluid cells [58]	DMEM/F12 + 10% FBS + 1 ng/mL bFGF + 0.1 mM nonessential amino acids + 1 mM L-glutamine + 0.1 mM *β*-mercaptoethanol	Superior immunomodulatory capability; inhibits the proliferation; activation of NK cells

Neonatal dermal fibroblasts [59]	(1) Cardiac myocyte culture medium (CARM) + SB-203580 to form embryoid bodies (2) Embryoid bodies plated, adherent growth, resulting in iMSC	Outstanding angiogenic activity; enhanced fibroblast migration ability

Adult dermal fibroblasts [26]	DMEM low glucose medium + 10% FBS + 2 mM L-glutamine	Higher cell proliferation rate

Urine cells and amniotic fluid cells [60]	*α*-MEM + 10% FBS + 50 *μ*M ascorbic acid 2-phosphate + L-glutamine + nonessential amino acids	Robust immunomodulatory capability; suppresses monocyte differentiation into dendritic cells; inhibits dendritic cell function

Blood cells [36]	(1) mTeSR1 + 10 *μ*M SB-431542 (2) ESC–MSC culture medium: KO DMEM + KOSR + NEAA + L-glutamine + *β*-mercaptoethanol + bFGF + EGF + SB-431542	Increased proliferative capacity; faster proliferation rate enhanced safety

Bone marrow mesenchymal stem cells [52]	(1) iPS culture: matrigel coating + mTeSR2 medium (2) iMSC differentiation: DMEM medium + 5 ng/mL bFGF + 0.1 mM NEAA + 1% glutamax + 10 *μ*M SB431542	Stronger proliferative capacity; longer telomere length; provides stronger support for hematopoietic stem cell proliferation

**Table 2:** iPSC-derived cellular products undergoing clinical research in China.

Manufacturer	Product	Cell source	Indications	Clinical stage
Allife medicine	“ALF201” injectable solution	iPSC-derived allogeneic endothelial progenitor cells (EPCs)	Atherosclerotic ischemic stroke	Phase I
Nuwacell	“NCR100” injectable solution	iPSC-derived mesenchymal stromal cells (MSCs)	Knee osteoarthritis	Phase I
	“NCR300” injectable solution	iPSC-derived natural killer cells (NK)	Myelodysplastic syndrome (MDS)	Phase I
Help therapeutics	“HiCM-188” injectable solution	iPSC-derived cardiomyocytes	Severe congestive heart failure	Phase I
Hopstem biotech	“hNPC01” injectable solution	iPSC-derived human forebrain neural precursor cells	Ischemic stroke hemiplegia sequelae	Phase I

## Data Availability

The authors confirm that all the data supporting the findings of this study are presented within the article. If any further information is required, then it may be provided upon reasonable request.
